# Clinical impact of OCT-derived suboptimal stent implantation parameters and definitions

**DOI:** 10.1093/ehjci/jead172

**Published:** 2023-07-18

**Authors:** Enrico Romagnoli, Francesco Burzotta, Rocco Vergallo, Laura Gatto, Giuseppe Biondi-Zoccai, Vito Ramazzotti, Flavio Biccirè, Simone Budassi, Carlo Trani, Ziad Ali, Gregg W Stone, Francesco Prati

**Affiliations:** Fondazione Policlinico Universitario A. Gemelli IRCCS, Rome, Italy; Fondazione Policlinico Universitario A. Gemelli IRCCS, Rome, Italy; Università Cattolica del Sacro Cuore, Largo Agostino Gemelli 8, 00168, Rome, Italy; Fondazione Policlinico Universitario A. Gemelli IRCCS, Rome, Italy; Azienda Ospedaliera San Giovanni Addolorata, Rome, Italy; Centro per la Lotta Contro L’Infarto—CLI Foundation, Rome, Italy; Department of Medical-Surgical Sciences and Biotechnologies, Sapienza University of Rome, Latina, Italy; Mediterranea Cardiocentro, Napoli, Italy; Azienda Ospedaliera San Giovanni Addolorata, Rome, Italy; Azienda Ospedaliera San Giovanni Addolorata, Rome, Italy; Centro per la Lotta Contro L’Infarto—CLI Foundation, Rome, Italy; Azienda Ospedaliera San Giovanni Addolorata, Rome, Italy; Centro per la Lotta Contro L’Infarto—CLI Foundation, Rome, Italy; Università Cattolica del Sacro Cuore, Largo Agostino Gemelli 8, 00168, Rome, Italy; St Francis Hospital & Heart Center, Roslyn, NY, USA; Cardiovascular Research Foundation, New York, NY, USA; The Icahn School of Medicine at Mount Sinai, Mount Sinai Heart and the Cardiovascular Research Foundation, New York, NY, USA; Azienda Ospedaliera San Giovanni Addolorata, Rome, Italy; Centro per la Lotta Contro L’Infarto—CLI Foundation, Rome, Italy; UniCamillus—Saint Camillus International University of Health Sciences, Rome, Italy

**Keywords:** optical coherence tomography, drug-eluting stent, clinical research, risk stratification

## Abstract

**Aims:**

Despite growing evidence supporting the clinical utility of optical coherence tomography (OCT) guidance during percutaneous coronary interventions (PCIs), there is no common agreement as to the optimal stent implantation parameters that enhance clinical outcome.

**Methods and results:**

We retrospectively examined the predictive accuracy of suboptimal stent implantation definitions proposed from the CLI-OPCI II, ILUMIEN-IV OPTIMAL PCI, and FORZA studies for the long-term risk of device-oriented cardiovascular events (DoCE) in the population of large all-comers CLI-OPCI project. A total of 1020 patients undergoing OCT-guided drug-eluting stent implantation in the CLI-OPCI registry with a median follow-up of 809 (quartiles 414–1376) days constituted the study population. According to CLI-OPCI II, ILUMIEN-IV OPTIMAL PCI, and FORZA criteria, the incidence of suboptimal stent implantation was 31.8%, 58.1%, and 57.8%, respectively. By multivariable Cox analysis, suboptimal stent implantation criteria from the CLI-OPCI II [hazard ratio 2.75 (95% confidence interval 1.88–4.02), *P* < 0.001] and ILUMIEN-IV OPTIMAL PCI [1.79 (1.18–2.71), *P* = 0.006] studies, but not FORZA trial [1.11 (0.75–1.63), *P* = 0.597], were predictive of DoCE. At long-term follow-up, stent edge disease with minimum lumen area <4.5 mm^2^ [8.17 (5.32–12.53), *P* < 0.001], stent edge dissection [2.38 (1.33–4.27), *P* = 0.004], and minimum stent area <4.5 mm^2^ [1.68 (1.13–2.51), *P* = 0.011] were the main OCT predictors of DoCE.

**Conclusion:**

The clinical utility of OCT-guided PCI might depend on the metrics adopted to define suboptimal stent implantation. Uncovered disease at the stent border, stent edge dissection, and minimum stent area <4.5 mm^2^ were the strongest OCT associates of stent failure.

## Graphical Abstract

**Graphical Abstract:**
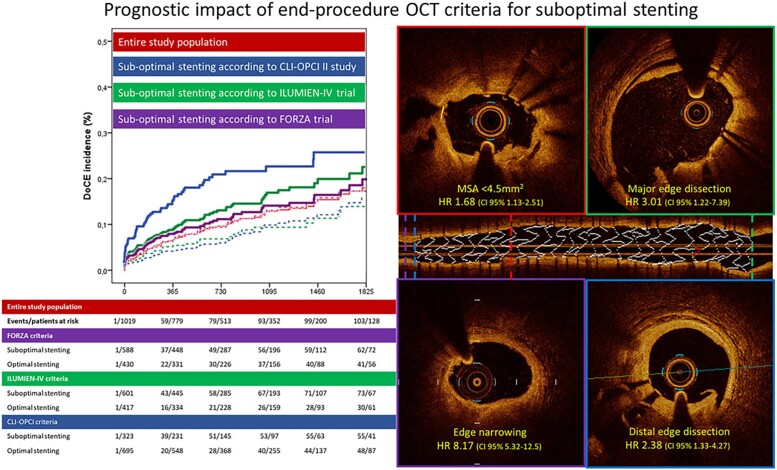

## Introduction

Improved clinical outcomes with imaging guidance during percutaneous coronary intervention (PCI) have been demonstrated in several randomized trials and registries.^1–8^ Nevertheless, the use of imaging guidance during PCI is still limited. Difficulties in image interpretation, uncertain clinical implications of specific imaging findings, and lack of evidence as to how to alter treatment based on these findings have contributed to this poor adoption.

To date, there is no formal agreement as to which criteria determine optimal stent implantation with either intravascular ultrasound (IVUS) or optical coherence tomography (OCT) guidance.^9^ As regards OCT, different stent implantation and optimization algorithms have gathered popularity. In particular, three recent studies assessed the clinical impact of OCT guidance during PCI: the Centro per la Lotta contro l’Infarto-Optimization of Percutaneous Coronary Intervention (CLI-OPCI II),^6^ the OPtical Coherence Tomography Guided Coronary Stent IMplantation Compared to Angiography: A Multicenter Randomized TriaL in PCI (ILUMIEN-IV OPTIMAL PCI),^10^ and the fractional flow reserve or optical coherence tomography guidance to revascularize intermediate coronary stenosis using angioplasty (FORZA)^11^ studies. In the present study, we aimed to compare the clinical performance of these three OCT-guided treatment protocols in the large all-comers patient population of the CLI-OPCI project registry, and to identify the specific OCT predictors of long-term drug-eluting stent (DES) failure.

## Methods

### Study design and endpoints

The first objective of the present study was to compare the different DES optimization strategies identified in the CLI-OPCI II registry,^6^ the ILUMIEN-IV OPTIMAL PCI trial,^10^ and the FORZA trial.^11^ In these studies, three different definitions of suboptimal stenting were proposed utilizing variable definitions and cut-offs (*Table 1*); in particular, the CLI-OPCI II criteria were retrospectively identified using end-procedural OCT images from an observational clinical registry, while those adopted in the two RCTs were pre-specified by investigators. Validation of each OCT-derived parameter indicated by the three studies as clinically relevant was assessed for their predictive accuracy in a large real-world patient cohort from the CLI-OPCI project.^8^ The CLI-OPCI project is an international multi-site observational registry (participating centres are listed in Appendix A) specifically designed study the relationships between end-procedural OCT findings and improved clinical outcomes (supplementary data). It included all consecutive PCIs performed with OCT guidance by centres expert in intravascular imaging, regardless of indication for imaging and operator’s interpretation. All final OCT acquisitions were collected and correlated with the risk of device-oriented cardiovascular events (DoCE) at long-term follow-up. A good quality final OCT pullback with a sufficient acquisition length to address the stented segments and at least 5 mm of the adjacent reference segments was the only mandatory inclusion criteria. Strengths of this registry were the large population size, the lack of pre-specified stent optimization algorithms, and the follow-up length. An additional objective of the study was to examine the specific stent-related OCT findings that predicted long-term adverse clinical outcomes.

**Table 1:** Criteria adopted for the definition of suboptimal stent implantation in the three studies

*CLI-OPCI II study*
In-stent minimum lumen area <4.5 mm^2^
Dissection thickness >200 μm at the distal stent edge
Distal or proximal reference narrowing (i.e. reference lumen area <4.5 mm^2^ in the presence of significant residual plaque adjacent to stent endings)
*FORZA trial*
Major stent strut malapposition (>350 μm or >200 μm for a length >600 μm)
Major stent underexpansion (in-stent minimal cross-section area of <75% of the reference lumen area)
Major stent edge dissection (>600 μm in length)
*ILUMIEN-IV OPTIMAL PCI trial*
Unacceptable stent expansion (minimal stent area <90% of the reference lumen area)
Major intra-stent plaque protrusion and/or thrombus (intraluminal mass protruding at least 0.2 mm within the luminal edge of a stent strut associated with unacceptable stent expansion, as defined above)
Untreated reference segment disease (disease with untreated minimum lumen area <4.5 mm^2^ within 5 mm from the stent edges)
Major edge dissection (≥60° of the circumference of the vessel at site of dissection and ≥3 mm in length)
Major stent malapposition (strut malapposition ≥200 μm associated with unacceptable stent expansion, as defined above)

The primary endpoint of the study was the correlation between the three different suboptimal stent implantation definitions and the incidence of DoCE during follow-up. The impact of each quantitative OCT parameter contributing to the definition of suboptimal stent implantation (*Table 1*) was then appraised. DoCE was defined as the composite of cardiac death, target vessel myocardial infarction (TV-MI, including peri-procedural MI defined as CK-MB >3 times the upper limit of normal), target lesion revascularization (TLR), or stent thrombosis (ST).^12^ All enrolled patients provided written informed consent for the index procedure and follow-up, and the CLI-OPCI project received ethical committee approval. A clinical events committee blinded to OCT findings adjudicated all adverse events according to the Academic Research Consortium guidelines.^13^ The project was supported by the Centro per la Lotta contro l’Infarto—Fondazione Onlus (CLI Foundation, Rome, Italy). The authors were solely responsible for the design, conduct, and analysis and publication of this study.

### OCT analyses and definitions

All OCT pullbacks were analysed off-line with a St. Jude review workstation (St Jude Medical, Inc., USA) by expert readers at a central core laboratory (Rome Heart Research).^14^ Definitions and quantitative cut-offs for OCT parameters were derived from the study designs (*Table 1*). In particular, we considered as markers of suboptimal stent implantation with the presence of: (i) ‘stent underexpansion’ expressed both as absolute in-stent minimum cross-sectional lumen area (MLA) < 4.5 mm^26^ or <75% relative to the average reference lumen area (RLA)^11^ or <90% relative to the closest RLA,^10^ (ii) ‘major acute stent malapposition’ defined as a malapposed stent distance from the vessel wall or plaque of >350 μm, or >200 μm for a length >600 μm or with unacceptable stent expansion, (iii) ‘major stent edge dissection’ defined as >200 μm in thickness, or length >600 μm in length, or ≥60° of the circumference of the vessel and ≥3 mm in length, (iv) ‘reference lumen narrowing’ defined as untreated residual plaque adjacent to a stent edge with lumen area <4.5 mm^2^,^6,10^ (v) and ‘intra-stent tissue prolapse’ defined as plaque/thrombus prolapse among stent struts into the vessel lumen.

### Statistical analysis

Discrete variables were reported as percentages, and continuous variables were reported as means (±standard deviation) if the data were normally distributed or medians (first–third quartile) otherwise. Bivariate analyses were performed using Student’s *t*-, Mann–Whitney *U*, and χ^2^ tests when appropriate. Adverse events were assessed on a per-patient hierarchical basis and summarized as Kaplan–Meier time-to-first event estimates. TLR and ST were analysed both on a per-patient level and a per-lesion level. Generalized mixed model analysis was performed to account for lesion and patient level clustering.

We explored the associations between individual end-procedural OCT parameters examined in the three studies and DoCE with Cox regression analysis. All variables in *Tables 2* and *3* were simultaneously forced into a non-parsimonious Cox regression model to identify independent outcome predictors and to calculate their adjusted hazard ratios (HRs) with associated 95% confidence intervals (CIs). Adjusted effect estimates of suboptimal stent implantation (defined as the presence of at least one parameter) were assessed from models in which the respective definitions were entered as covariates.

**Table 2:** Baseline patient characteristics and their adjusted relationships with device-oriented cardiovascular events during follow-up

	All patients (*n* = 1020)	Hazard ratio (95% CI)	*P*-value
Age (years)^a^	64 (56–71)	1.01 (0.98–1.03)	0.847
Female sex (%)	217 (21.3)	1.02 (0.57–1.82)	0.946
Left ventricle ejection fraction (%)^a^	56 (50–60)	0.97 (0.94–0.98)	0.026
Hypertension (%)	708 (69.4)	0.85 (0.48–1.49)	0.565
Hypercholesterolaemia (%)	635 (62.3)	0.96 (0.58–1.59)	0.868
Smoking (%)	324 (31.8)	1.58 (0.88–2.84)	0.122
Family history of CAD (%)	336 (32.9)	0.77 (0.45–1.34)	0.358
Diabetes mellitus (%)	232 (22.7)	2.12 (1.23–3.64)	0.007
CKD (GFR <60 mL/min/1.73 m^2^) (%)	130 (12.7)	1.56 (0.85–2.86)	0.153
Multi-vessel disease (%)	546 (53.5)	1.19 (0.71–1.99)	0.512
Prior MI (%)	223 (21.9)	0.99 (0.55–1.79)	0.968
Prior coronary revascularization (%)	339 (33.2)	1.82 (1.06–3.18)	0.036
Acute coronary syndrome (%)	568 (55.7)	1.35 (0.81–2.24)	0.252
STEMI (%)	258 (25.3)	1.11 (0.62–1.97)	0.731
NSTEMI (%)	142 (13.9)	2.27 (1.27–4.08)	0.006
Unstable angina (%)	168 (16.5)	0.65 (0.33–1.27)	0.207
Stable angina (%)	452 (44.3)	0.74 (0.48–1.24)	0.252

CAD, coronary artery disease; CKD, chronic kidney disease; GFR, glomerular filtration rate; DoCE, device-oriented cardiovascular events; MI, myocardial infarction; NSTEMI, non-ST-segment elevation MI; STEMI, ST-segment elevation MI.

^a^Expressed as median and interquartile range.

**Table 3:** Incidence of end-procedural OCT parameters used to define suboptimal stent implantation and their relationships with device-oriented cardiovascular events during follow-up

	All lesions (*n* = 1190)	Hazard ratio (95% CI)	*P*-value
*CLI-OPCI II criteria*			
Suboptimal stent implantation (%)	378 (31.8)	2.75 (1.88–4.02)	<0.001
Stent underexpansion (MSA <70% of RLA) (%)	261 (21.9)	1.38 (0.91–2.11)	0.132
Minimum in-stent lumen area <4.5 mm^2^ (%)	286 (24.0)	1.68 (1.13–2.51)	0.011
Edge dissection >200 μm in thickness(%)	143 (12.0)	1.69 (1.04–2.77)	0.035
Distal edge (%)	74 (6.2)	2.38 (1.33–4.27)	0.004
Proximal edge (%)	79 (6.6)	0.81 (0.35–1.84)	0.609
Intra-stent plaque/thrombus protrusion >500 μ (%)	314 (26.4)	1.49 (0.98–2.7)	0.066
Reference narrowing (%)^§^	76 (6.4)	8.17 (5.32–12.53)	0.001
Distal narrowing (%)	59 (5.0)	6.98 (4.32–11.26)	<0.001
Proximal narrowing (%)	23 (1.9)	7.33 (3.68–14.63)	<0.001
Malapposition >200 μ (%)	622 (52.3)	1.08 (0.74–1.58)	0.698
*ILUMIEN-IV OPTIMAL PCI criteria*			
Suboptimal stent implantation (%)	691 (58.1)	1.79 (1.18–2.71)	0.006
Stent underexpansion (MSA <90% of RLA) (%)	637 (53.5)	0.94 (0.64–1.38)	0.756
Major edge dissection (>60° and/or >3 mm in length) (%)	20 (1.7)	3.01 (1.22–7.39)	0.016
Reference narrowing (%)^a^	76 (6.4)	8.17 (5.32–12.53)	<0.001
Intra-stent plaque/thrombus protrusion >200 μm and MSA <90% of RLA (%)	122 (10.3)	0.73 (0.35–1.50)	0.388
Malapposition >200 μ and MSA <90% of RLA (%)	370 (31.1)	0.85 (0.56–1.30)	0.464
*FORZA criteria*			
Suboptimal stent implantation (%)	688 (57.8)	1.11 (0.75–1.63)	0.597
Stent underexpansion (MSA <75% of RLA) (%)	362 (30.4)	1.05 (0.70–1.57)	0.824
Major Edge Dissection (>600 μm length) (%)	179 (15.0)	1.30 (0.79–2.17)	0.306
Malapposition >350 μm or >200 μm for 600 μm (%)	373 (31.3)	0.96 (0.63–1.49)	0.837

MSA, minimum stent area; RLA, reference lumen area.

^a^Defined as reference lumen area <4.5 mm^2^ in the presence of significant plaque.

*c*-Statistics and areas under the curves were computed from the receiver-operating characteristic curves to compare the discriminative capacity of suboptimal stent implantation definitions according to the CLI-OPCI II, ILUMIEN-IV OPTIMAL PCI, and FORZA studies.^15^ The analyses were done and reported in accordance with the transparent reporting of a multivariable prediction model for individual prognosis or diagnosis (TRIPOD) statement.^16^

A two tailed, *P*-value of <0.05 was established as the level of statistical significance for all tests, without multiplicity adjustment. Data were analysed using the statistical software Stata 15.1 (StataCorp, College Station, TX).

## Results

**Figure 1:**
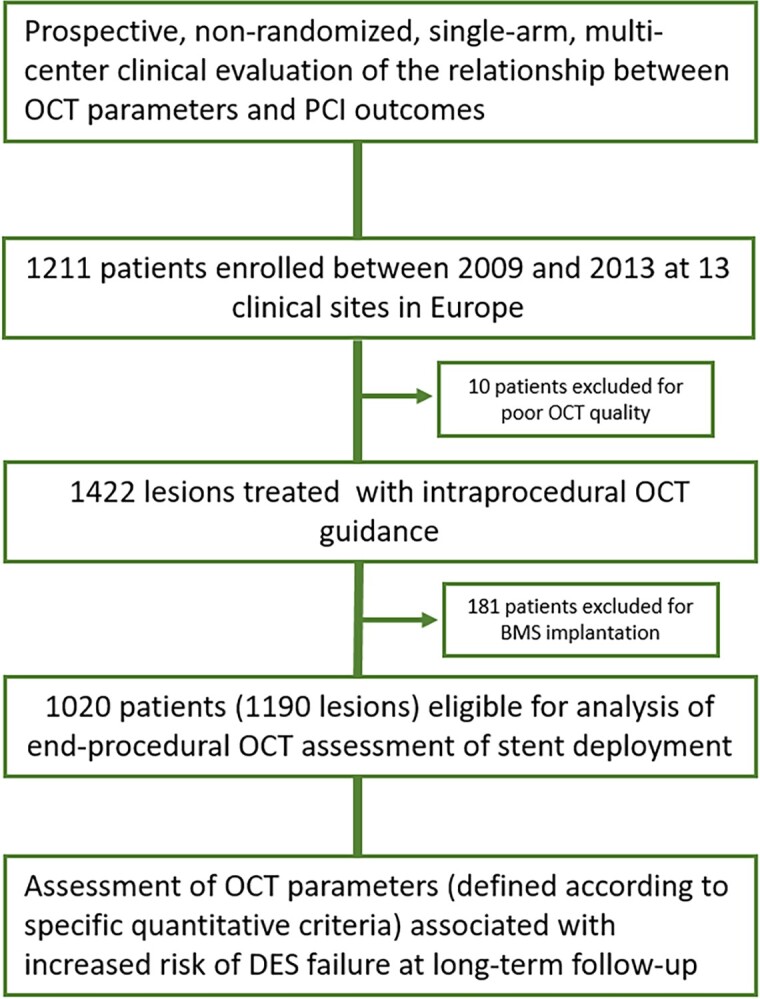
Study flow chart.

In this analysis, we included from the CLI-OPCI project population 1020 patients undergoing OCT-guided second-generation DES implantation in 1190 lesions (*Figure 1*). *Table 2* summarizes the main clinical characteristics of the study population. Median patient age was 64 [interquartile range (IQR) 56–7] years with 21.3% females; 55.7% of patients presented with an acute coronary syndrome including acute MI in 25.3% of cases. Most treated lesions were complex (79.8% modified Ellis class B2/C) with multiple overlapping stents required in 22.8% of cases. Nonetheless, a satisfactory angiographic result (visually-estimated residual stenosis <30% and TIMI 3 flow) was obtained in 97.8% of procedures.

### OCT findings

The incidence of suboptimal stent implantation criteria used in the three studies is summarized in *Table 3*. End-procedural OCT assessment demonstrated relative stent underexpansion exceeding 10%, 25%, and 30% of the RLA in 53.5%, 30.4%, and 21.9% of stents, respectively; absolute stent underexpansion (in-stent MLA <4.5 mm^2^) in 24.0% of stents; edge dissection with thickness >200 μm in 12.0%, with length >600 μm in 15.0%, and with angle >60° and/or >3 mm in length in 1.7%; acute residual stent malapposition >200 μm in 52.3%, >200 μm associated with minimum stent area (MSA) <90% of RLA in 31.1%, and >350 μm and/or >200 μm for 600 μm in length in 31.3%; intra-stent plaque/thrombus protrusion >500 μm in 26.4%, >200 μm associated with MSA <90% of RLA in 10.3%; and reference lumen narrowing (defined as residual plaque >70% adjacent to a stent edge with lumen area <4.5 mm^2^) in 6.4% of cases. Cumulatively, the incidences of suboptimal stent implantation were 31.8% according to CLI-OPCI II criteria, 58.1% according to ILUMIEN-IV OPTIMAL PCI criteria, and 57.8% according to FORZA criteria.

### Outcome predictors

During median follow-up of 809 (IQR 414–1376) days, 10.5% of the patients experienced a DoCE including 2.7% cardiac death, 6.6% TV-MI, 7.7% TLR, and 1.8% definite or probable stent thrombosis. Median time-to-DoCE were 809 days (IQR 384–1343), with 55.1% of adverse events occurring within the first year after the procedure (*Table 4*).

**Table 4:** Clinical outcomes during follow-up

	All population (*n* = 1020)
Device-oriented cardiovascular events (%)	107 (10.5)
Cardiac death (%)	28 (2.7)
Target vessel MI (%)	67 (6.6)
Target lesion revascularization (%)	
Patient level	76 (7.5)
Lesion level	92 (7.7)
Target vessel revascularization (%)	
Patient level	90 (8.8)
Lesion level	110 (9.2)
Stent thrombosis, definite or probable (%)	
Patient level	21 (2.1)
Lesion level	22 (1.8)
Days of follow-up [median (interquartile range)]	809 (414–1376)

**Figure 2:**
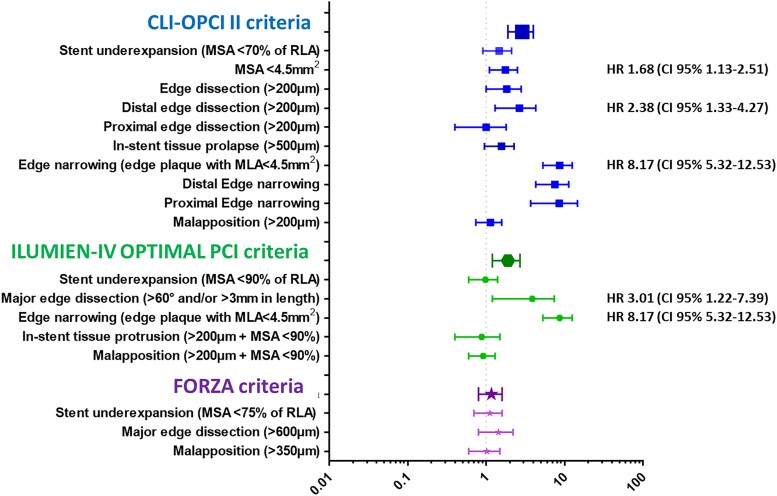
Predictive hazards of all OCT parameters tested in the CLI-OPCI II, ILUMIEN-IV OPTIMAL PCI, and FORZA studies.

**Figure 3:**
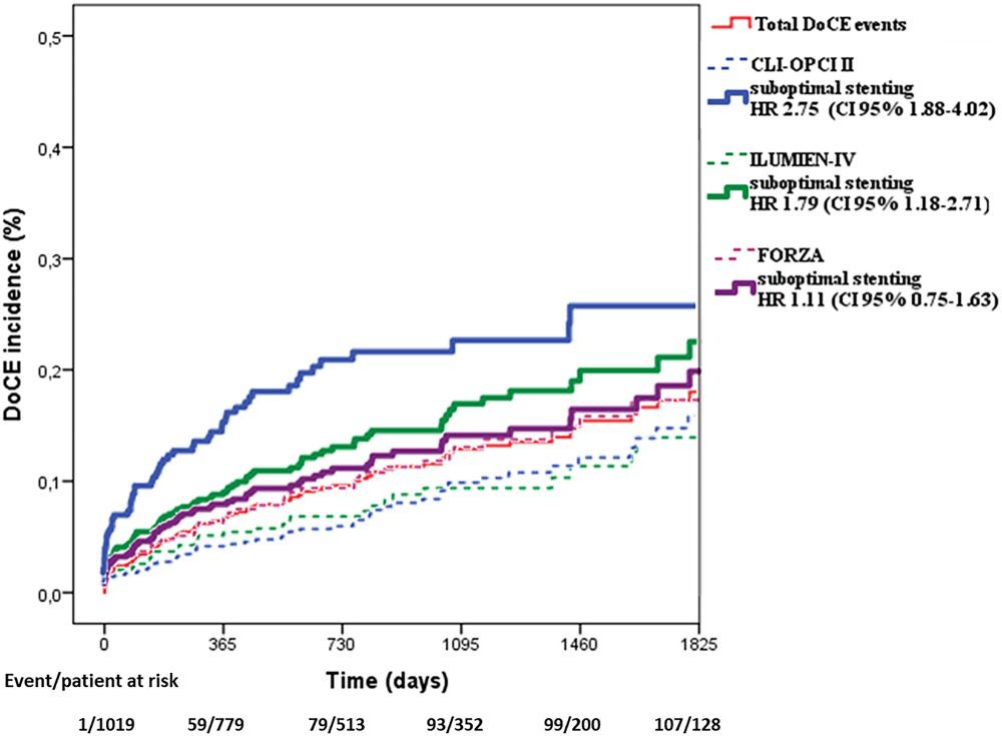
Clinical outcomes of suboptimal (full lines) and optimal stent implantation (dotted lines) as defined according to CLI-OPCI II, ILUMIEN-IV OPTIMAL PCI, and FORZA criteria.

**Figure 4:**
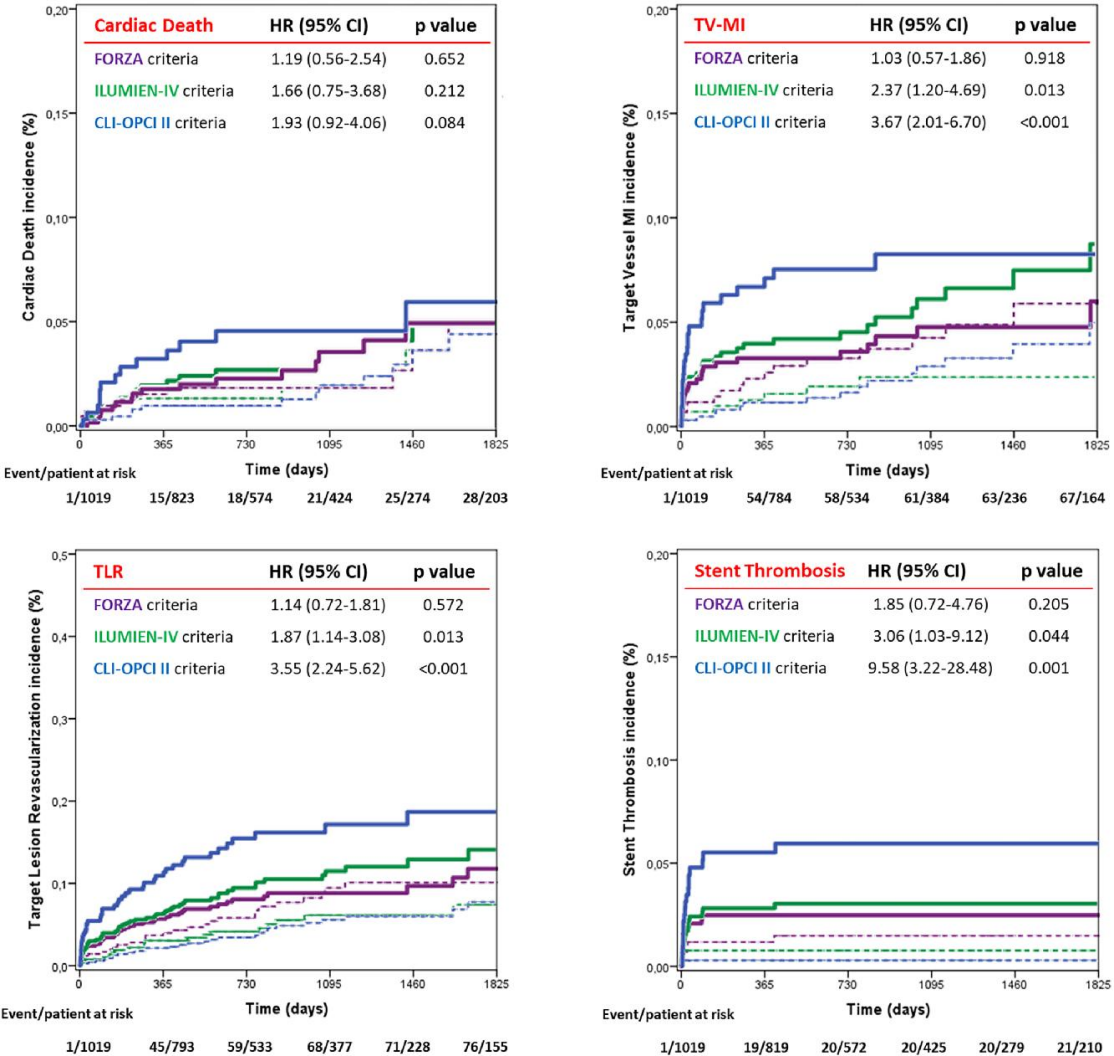
Time-to-event curves for individual device-oriented cardiovascular events (DoCE) components in patients with (full lines) and without (dotted lines) suboptimal stent implantation according to the different study protocols.

By multivariable analysis non-ST-segment elevation MI diagnosis, diabetes mellitus, prior coronary intervention, and left ventricle ejection fraction (LVEF) were independent clinical predictors of DoCE (*Table 2*). End-procedural OCT characteristics associated with DoCE were in-stent MLA <4.5 mm^2^, dissection thickness >200 μm at distal stent edge or dissection >60° and/or >3 mm in length, and reference lumen narrowing <4.5 mm^2^ in presence of significant plaque (*Table 3*). The definitions of suboptimal stent implantation from the CLI-OPCI II study (HR 2.75, 95% CI 1.88–4.02, *P* < 0.001) and the ILUMIEN-IV OPTIMAL PCI study (HR 1.79, 95% CI 1.18–2.71, *P* = 0.006) were predictive for DoCE whereas the suboptimal stent implantation criteria from the FORZA trial (HR 1.11, 95% CI 0.75–1.63, *P* = 0.597) were not (*Figures 2* and *3*). The predictive value of these definitions was confirmed after correction for clinical and procedural variables: HR 1.70, 95% CI 1.13–2.79, *P* = 0.038 for the ILUMIEN-IV OPTIMAL PCI definition, HR 2.31, 95% CI 1.48–3.67, *P* < 0.001 for the CLI-OPCI II, and HR 1.05, 95% CI 0.65–1.66, *P* = 0.863 for FORZA. In particular, the criteria for suboptimal stent implantation used in CLI-OPCI II had better discrimination [*c*-index 0.63 (95% CI 0.57–0.68)] than the criteria used in both the ILUMIEN-IV OPTIMAL PCI [*c*-index 0.56 (95% CI 0.51–0.62), *P* = 0.026] and FORZA [*c*-index 0.51 (95% CI 0.45–57), *P* < 0.001] trials. Performance of the three models for individual DoCE components is reported in supplementary data and *Figure 4*.

## Discussion

This study compared the performance of varying criteria and parameters of OCT-based DES optimization from a large real-world patient registry with more than 2 years of follow-up. The main findings of this study are the following: (i) the CLI-OPCI II and ILUMIEN IV OCT-based stent optimization criteria were independently predictive of stent failure, with the CLI-OPCI II definition having the greatest discrimination; and (ii) several OCT parameters of suboptimal stent implantation were also strongly related to DoCE during follow-up; among these, final in-stent MLA <4.5 mm^2^, (distal) stent edge dissection (>200 μm in thickness or >60° and >3 mm in length), and reference edge narrowing with a RLA <4.5 mm^2^ showed the strongest predictive associations for DoCE.

There is a growing evidence supporting the benefit of intravascular imaging guidance during PCIs in terms of both acute procedural results (i.e. MSA) and clinical outcome.^2^ Indeed, both American and European current guidelines recognized the clinical value of IVUS or OCT in complex PCI settings.^17,18^ In this context, IVUS has the more solid evidence based on several RCTs conducted with modern DES platforms,^19,20^ and on meta-analyses demonstrating the improvement of hard clinical endpoints such as cardiovascular mortality.^21^ The main advantages of IVUS in comparison to standard angiography guidance could be coarsely summarized in a greater MSA (i.e. media-to-media approach for stent size selection), reduced major stent struts malapposition, and less stent edge dissection left untreated.

Compared to IVUS, OCT is able to provide a more detailed analysis of coronary plaque morphology^22,23^ and plaque/stent interactions^24,25^ but, the clinical role of these parameters is still undetermined. Indeed, the evidence supporting OCT use in PCIs is limited to observational registries with only few randomized trials focused on surrogate endpoints (i.e. improved post-PCI flow fractional reserve (FFR)) and underpowered for the clinical outcome.^26–28^

Thus, OCT is commonly used as a tool to confirm lesion severity in uncertain conditions, to determine the magnitude of coronary calcification, and to select the optimal device size.^29,30^ Nevertheless, OCT approach is largely based on quantitative criteria borrowed from the validated IVUS experience.

The few trials comparing IVUS and OCT performance in PCIs showed similar results in terms of stent expansion and mid-term clinical outcome,^31–33^ nevertheless, using OCT quantitative measurements is generally 10–20% smaller than IVUS,^34^ plaque burden is often not assessable,^35^ while also trivial stent malapposition or edge dissection can be detected. These important differences between IVUS and OCT explain the need for a specific and standardized OCT-based definition of optimal stent implantation.

In this view, recently algorithmic approaches to OCT utilization have been developed taking into account not only stent expansion and reference vessel diameters but also the detection and quantitative assessment of parameters such as strut malapposition,^36^ in-stent tissue protrusion,^7^ and residual stent edge dissection^37,38^ findings that are often missed by angiography and may contribute to the mechanisms subtending DES failure.^6–8,11^ Nevertheless, most of studies assessing the utility if OCT guidance during PCI are limited by important lesion selection bias, retrospective data collection, and heterogeneity of OCT metrics considered. As a result, consensus regarding the best definition of OCT-based suboptimal stent implantation is lacking.

Three large studies have attempted to fill this void by proposing algorithms for optimal OCT-guided stent implantation on the basis of quantitative criteria: the CLIO-PCI II registry,^6^ a retrospective registry identifying the end-procedural OCT parameters associated with an increased risk of DoCE at 1 year follow-up; the ongoing large-scale ILUMIEN-IV OPTIMAL PCI study, a randomized trial designed to demonstrate the superiority of OCT-guided vs. angiography-guided DES implantation in patients with high-risk clinical characteristics and/or complex angiographic lesions,^10^ and the FORZA study, a randomized controlled trial that demonstrated an advantage of OCT guidance for the treatment of intermediate lesions compared to FFR guidance in terms of major adverse cardiac events or angina.^11^

**Figure 5:**
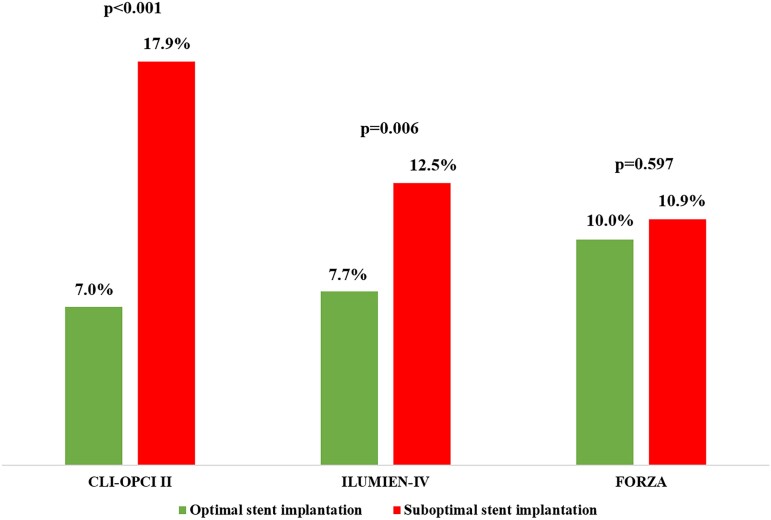
DoCE incidence according to the different definitions of suboptimal stent implantation proposed in the CLI-OPCI II, ILUMIEN-IV OPTIMAL PCI, and FORZA studies.

In our study population, including only patients treated with second-generation DES and long-term follow-up, the CLI-OPCI II and ILUMIEN-IV OPTIMAL PCI optimal stent implantation criteria, but not the FORZA criteria, were predictive for stent failure risk (*Figure 5*). Notably, OCT-defined suboptimal stent implantation in these trials was confirmed as an independent predictor of DoCE after correction for the other clinical and procedural variables. These data are in line with the results of the recent RENOVATE-COMPLEX-PCI trial,^33^ showing a durable lower incidence of target vessel failure with intravascular guidance when compared to angiography guidance (7.7% vs. 12.3% after 2 years), with a more favourable outcome in patients who had stent optimization according to the pre-specified IVUS or OCT criteria (6.0% vs. 8.9%).

**Figure 6:**
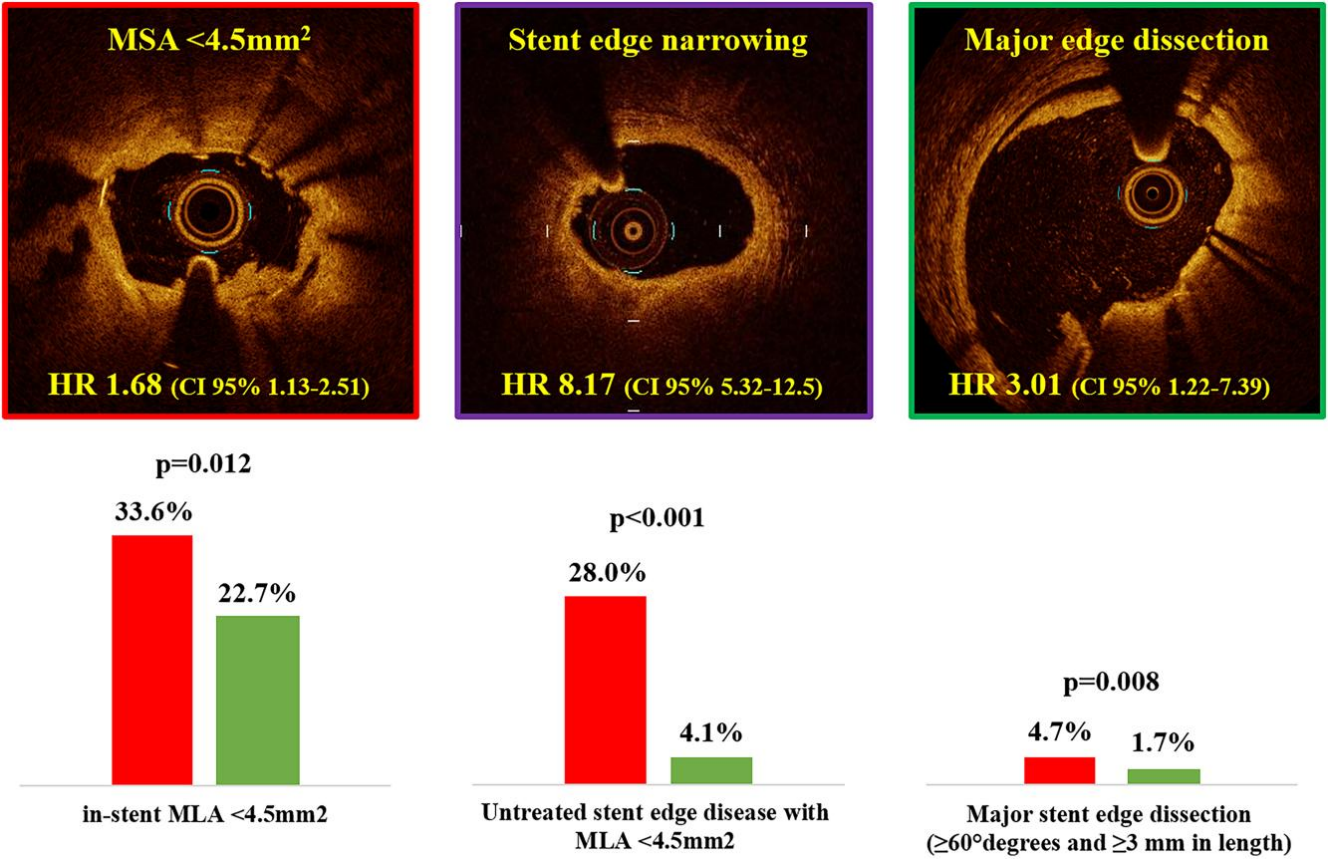
End-procedural OCT findings associated with DES failure.

These data support the potential clinical utility of OCT guidance during PCI equal to IVUS, although outcomes from the ongoing large-scale ILUMIEN-IV OPTIMAL PCI randomized trial^10^ are necessary before drawing definitive conclusions regarding specific parameters and cut-offs (*Figures 5* and *6*).

While these studies proposed an algorithm to guide stent implantation using quantitative OCT parameters (*Table 1*), of note, the methodology and the criteria adopted to define suboptimal stent implantation in these three studies varied. For example, stent expansion was measured as an absolute value in CLI-OPCI II, but as relative to the mean reference vessel area in the ILUMIEN-IV OPTIMAL PCI and FORZA trials. The different performance of these varying criteria emphasizes the importance of specific definitions and perhaps procedural guidance techniques to achieve these metrics if clinical outcomes are to be improved. Indeed, the rate of suboptimal stent implantation observed (ranging from 31.8% to 58.1%) and its correlation with stent failure give a measure of improvement opportunities related to OCT- guidance.

The analysis of individual OCT parameters indicated absolute in-stent MLA <4.5 mm^2^, dissection at the stent edge, and reference vessel disease (i.e. geographic miss) with residual lumen area <4.5 mm^2^ as the best predictors of stent failure (*Figure 6*). Thus, albeit the intravascular imaging basically guides the operator to obtain a larger MSA, stent expansion does not seem the only parameter impacting of PCI outcome. Moreover, we cannot exclude that the impact of a single OCT parameter could vary in different anatomical or clinical contexts.^7^ For example, the relative risk derived from stent underexpansion could be different in culprit lipid-rich plaques in acute coronary syndromes compared to stable calcified lesions. Similarly, the impact of stent malapposition might also depend on the absolute MSA or anatomical features, resulting innocuous in large vessels, but detrimental in smaller vessels or bifurcation lesions.

### Study limitations

Our analyses were applied to the data from a retrospective registry including patients with few exclusion criteria, including those with chronic and acute coronary syndromes, different stents, and a variety of treatment approach (including how the information derived from OCT was used to modify treatment). We cannot exclude bias in terms of patient and lesion selection for OCT use; the choice to leave some OCT findings untreated may have been conditioned by the clinical context more than individual practice. Nevertheless, the exclusive use of end-procedural OCT pullback and the absence of pre-specified optimal stent definition afforded a broad-based examination of OCT use in daily practice across different operators and sites facilitating the present analysis relating differing stent parameters to clinical outcomes.

Finally, the best performance of CLI-OPCI II criteria in this study could be related to a partial overlap in terms of patients and operators. Indeed, albeit we included in this analysis only patients with second-generation DES implantation from larger and different population, we cannot exclude that operators from centres involved in the CLI-OPCI II study (five centres out of the actual 13 involved in the CLI-OPCI project) were more prone to pursuit that standards.

## Conclusions

This present study supports the prognostic utility of several OCT parameters after DES implantation and suggests that OCT-guided stent optimization to improve these indices might enhance clinical outcomes. Specifically, final in-stent MLA <4.5 mm^2^, (distal) stent edge dissection, and reference edge narrowing with a RLA <4.5 mm^2^ showed the strongest predictive associations for DoCE. Whether striving to optimize these parameters improves PCI outcomes of DES implantation in high-risk patients and complex lesions will be examined in the large-scale ongoing ILUMIEN-IV OPTIMAL PCI trial.

## Supplementary data

Supplementary data are available at *European Heart Journal - Cardiovascular Imaging* online.

## Supplementary Material

jead172_Supplementary_Data

## Data Availability

The data underlying this article will be shared on reasonable request to the corresponding author.
