# Supplementary figures and images for: Clinical impact of OCT-derived suboptimal stent implantation parameters and definitions
Source: Eur Heart J Cardiovasc Imaging. 2023 Jul 18;25(1):48–57. doi: 10.1093/ehjci/jead172 (PMC10735315; doi:10.1093/ehjci/jead172)

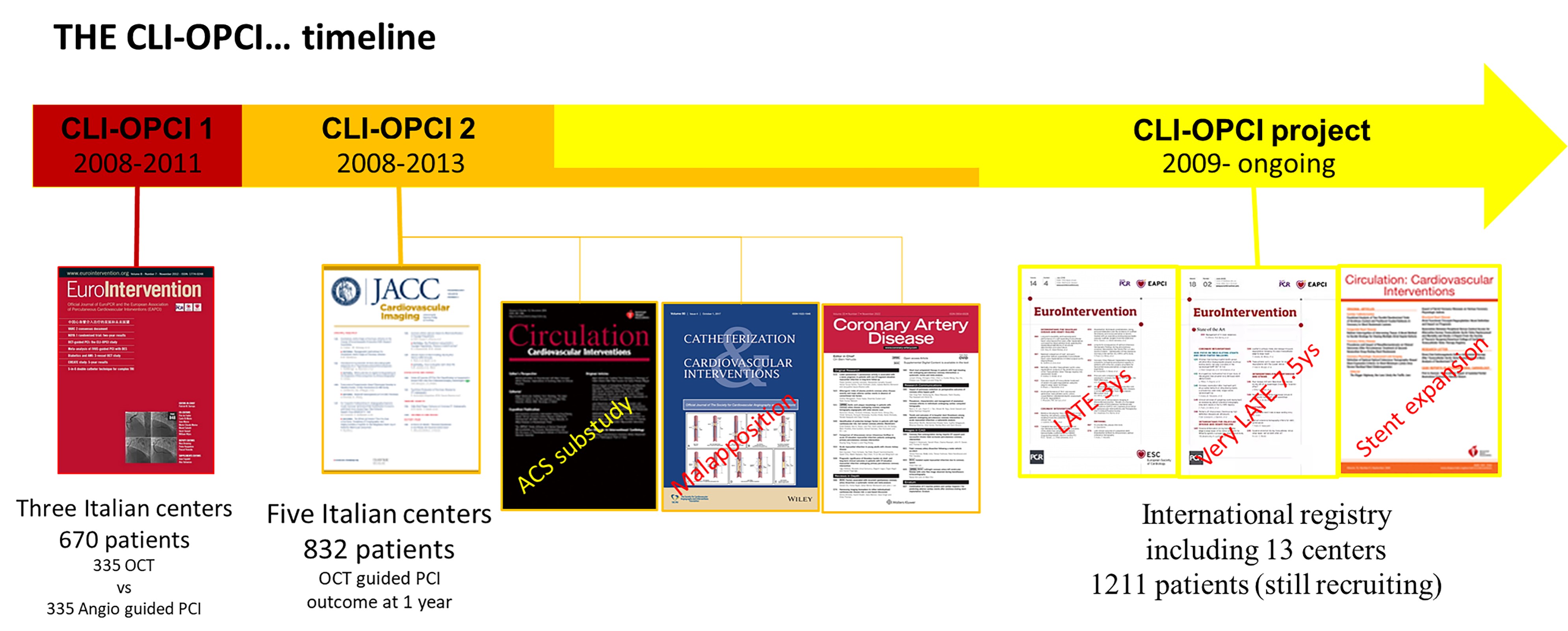

Supplement: jead172_Supplementary_Data [file jead172_supplementary_data.jpeg]
